# Similarities between the biochemical composition of jellyfish body and mucus

**DOI:** 10.1093/plankt/fbab091

**Published:** 2022-01-21

**Authors:** Nathan Hubot, Sarah L C Giering, Cathy H Lucas

**Affiliations:** National Oceanography Centre, Waterfront Campus, Southampton SO14 3ZH, UK; Ocean and Earth Science, University of Southampton, National Oceanography Centre, Waterfront Campus, Southampton SO14 3ZH, UK; National Oceanography Centre, Waterfront Campus, Southampton SO14 3ZH, UK; Ocean and Earth Science, University of Southampton, National Oceanography Centre, Waterfront Campus, Southampton SO14 3ZH, UK

**Keywords:** protein, lipid, carbohydrate, nitrogen, carbon

## Abstract

Recognition of the importance of jellyfish in marine ecosystems is growing. Yet, the biochemical composition of the mucus that jellyfish constantly excrete is poorly characterized. Here we analyzed the macromolecular (proteins, lipids and carbohydrates) and elemental (carbon and nitrogen) composition of the body and mucus of five scyphozoan jellyfish species (*Aurelia aurita*, *Chrysaora fulgida*, *Chrysaora pacifica*, *Eupilema inexpectata* and *Rhizostoma pulmo*). We found that the relative contribution of the different macromolecules and elements in the jellyfish body and mucus was similar across all species, with protein being the major component in all samples (81 ± 4% of macromolecules; 3.6 ± 3.1% of dry weight, DW) followed by lipids (13 ± 4% of macromolecules; 0.5 ± 0.4%DW) and carbohydrates (6 ± 3% of macromolecules; 0.3 ± 0.4%DW). The energy content of the jellyfish matter ranged from 0.2 to 3.1 KJ g^−1^ DW. Carbon and nitrogen content was 3.7 ± 3.0 and 1.0 ± 0.8%DW, respectively. The average ratios of protein:lipid:carbohydrate and carbon:nitrogen for all samples were 14.6:2.3:1 and 3.8:1, respectively. Our study highlights the biochemical similarity between the jellyfish body and mucus and provides convenient and valuable ratios to support the integration of jellyfish into trophic and biogeochemical models.

## INTRODUCTION

Jellyfish (cnidarian medusae and ctenophores) can affect the marine food chain and biogeochemical cycles by converting large amounts of organic matter from low trophic levels (e.g. primary producers) into gelatinous biomass at higher trophic levels (Condon et al., 2011). Up to 7% of the carbon assimilated by jellyfish is released in the environment in the form of mucus (Hansson and Norrman, 1995), which has been suggested to play an important role in carbon cycling (Hansson and Norrman, 1995; Condon et al., 2011; Tinta et al., 2021). Yet, jellyfish mucus has received little attention. For examples, the rates of mucus excretion, mucus composition and its fate in the ecosystem are not well understood. To date, the biochemical composition of jellyfish mucus has been analyzed for only three species (two medusae and one ctenophore) using different methods of collection and analysis (Ducklow and Mitchell, 1979; Condon et al., 2011); thus characterization of jellyfish mucus remains largely unclear (Tinta et al., 2021). In the context of expected jellyfish population increase, it is increasingly important to include jellyfish in energy flux models and ecosystem studies (Ramondenc et al., 2020). The lack of knowledge of jellyfish mucus composition challenges our ability to understand and model its role in the marine ecosystem.

Cnidarian mucus is predominantly composed of water, ~95% of its wet mass, with the remaining 5% composed of glycoproteins (~3%) and other molecules (~2%) such as antibodies, peptides, lipids, nucleic acids and inorganic salts such as sodium chloride (Stabili et al., 2015; Bakshani et al., 2018). The glycoproteins dictate the biophysical properties of the mucus, namely its viscosity and elasticity (Bansil and Turner, 2018), allowing it to lubricate and protect the underlying epithelia as well as to entrap, entrain and transport particles to the digestive pouches (Bakshani et al., 2018). Jellyfish can excrete large amounts of mucus in different situations, including under stress, during digestion and during reproduction (Patwa et al., 2015). Once released into the environment, jellyfish mucus is quickly metabolized by bacteria, creating major shifts in microbial assemblages and shunting carbon toward bacterial respiration (Condon et al., 2011). The fast remineralization of jellyfish mucus potentially releases nutrients to the environment with elemental ratios reflecting its composition.

To our knowledge, the macromolecular (protein/lipid/carbohydrate) composition of jellyfish mucus has been measured only once for the scyphomedusa *Aurelia aurita* (proteins = 73%, lipids = 27% and carbohydrates = 5%; Ducklow and Mitchell, 1979), which appeared comparable to the macromolecular composition of the species’ whole body. Thus, we hypothesize that the consistency in macromolecular composition of the body and the mucus of jellyfish medusae is ubiquitous and can be found in other species of scyphomedusae. In addition, we expect the elemental composition of the mucus to also reflect the elemental composition of the body. To test our hypotheses, we performed macromolecular and elemental analyses on the jellyfish body and mucus of five scyphomedusae species (*A. aurita*, *Chrysaora fulgida*, *Chrysaora pacifica*, *Eupilema inexpectata* and *Rhizostoma pulmo*).

## MATERIALS AND METHODS

Adult medusae of five scyphomedusae species (*A. aurita*, *C. fulgida*, *C. pacifica*, *E. inexpectata* and *R. pulmo*) were collected, following the protocol by Hubot et al. (2021), from Horsea Lake (UK), Walvis Bay (Namibia), the London Aquarium (UK), Port Elizabeth (South Africa) and the Isle of Portland (UK), respectively. The identity of *R. pulmo*, which is typically of the Mediterranean and adjacent seas (Holst et al., 2007), was confirmed genetically (Ramšak, pers. comm.) Following transfer to the laboratory, specimens were rinsed with filtered seawater (0.7 μm) and the body tissue and mucus samples were collected. Mucus samples were collected for all five species, while body samples were only available for four of the species (*C. pacifica* missing). Mucus was collected by placing the medusae in an empty clean container. The stress caused by the absence of seawater induced the production of mucus and its accumulation in the container. This aggressive approach of mucus collection has the advantage of quickly obtaining dense mucus material, although we acknowledge that stress-induced mucus might differ slightly compared with mucus produced under “natural” conditions. When a minimum of 50 mL of mucus was produced (after 1–15 min depending on the size of the jellyfish), the medusae were removed and the mucus transferred to a 50 mL polypropylene centrifuge tube via a clean glass funnel. For small medusae (*A. aurita*) the whole body was frozen, whereas for large specimens (*C. fulgida*, *E. inexpectata* and *R. pulmo*) a pie section of the medusae body (½ for *C. fulgida* and ¼ for *E. inexpectata* and *R. pulmo*) containing all organs and tissue (including umbrella, gonads and arms) in the same proportion as the full body was sliced off and stored at −20°C. Double-bagged frozen samples were carefully crushed using a hammer and lyophilized using a freeze drier. After lyophilization, samples were ground into a fine powder using a clean mortar and pestle and kept at −20°C.

Total lipids were extracted using a single-step extraction method based on the chloroform–methanol solvent system following the protocol by Axelsson and Gentili (2014; see details in Supplementary Information (SI)). Total proteins were measured using a modification of the Lowry assay by Gerhardt et al. (1994; see details in SI). Total carbohydrates were measured following the protocol by DuBois et al. (1956, see details in SI). The carbon and nitrogen content of the samples were measured using a CHNS Elemental Analyzer (Elementar Vario Micro Cube, see details in SI). Ash-free dry weight (AFDW, i.e. the organic portion of the dry weight) was calculated by measuring the ash weight (AW) following combustion at 400°C in a muffle furnace using an ultra-microbalance (Sartorius SE2, readability: 0.1 μg; see details in SI) and subtracting this value from the dry weight (DW; AFDW = DW – AW). All samples were measured in triplicate.

The energy content (EC) of the jellyfish body and mucus samples was calculated based on the macromolecule composition (protein, lipid and carbohydrate) and their mean combustion equivalents (Equation 1; Doyle et al., 2007). As significant amounts of bound water (~10% of DW) remain in medusae after drying (i.e. freeze-drying or drying at 50°C; Larson, 1986), the measurements were corrected for the dilution effect of the bound water by assuming a residual hydration of 11.7%DW (correction factor: 1.13). Hence, EC (kJ g^−1^) was calculated as:

*EC* = [(% protein × *a*) + (% lipid × *b*) + (% carbohydrate × *c*)] × *d*, (1)

where *a, b* and *c* are the gross energy values for protein, lipid and carbohydrate of gelatinous zooplankton (23.9, 39.5 and 17.5 kJ g^− 1^, respectively; Clarke et al., 1992), and *d* is the water of hydration correction factor of 1.13.

All biochemical measurements were normalized to the DW of the sample. The relationships between the macromolecular content (protein/lipid/carbohydrate) of the samples were modeled using linear regression and the differences between the linear regressions of mucus and body were investigated by analysis of covariance. All statistical tests were performed in R (version 4.0.3).

## RESULTS

The total of macromolecules (protein + lipid + carbohydrate) content was 7.3 ± 3.6 and 1.7 ± 0.9%DW for the jellyfish body and mucus, respectively. The amount of AFDW (total organic content) was 20.0 ± 3.9 and 12.9 ± 1.5%DW for body and mucus, respectively. Protein was the main component of the jellyfish body tissue (82 ± 4% of macromolecules; 6.0 ± 3.0%DW) and of the mucus (80 ± 4% of the macromolecules; 1.4 ± 0.8%DW), followed by lipids (body: 11 ± 3% of the macromolecules, 0.7 ± 0.4%DW; mucus: 14 ± 4% of the macromolecules, 0.2 ± 0.1%DW) and carbohydrates (body: 7 ± 4% of the macromolecules, 0.6 ± 0.4%DW; mucus: 6 ± 3%% of the macromolecules, 0.1 ± 0.1%DW). The jellyfish body tissue contained 6.2 ± 2.4%DW of carbon and 1.7 ± 0.6%DW of nitrogen, resulting in a C:N ratio of 3.6 ± 0.2. The jellyfish mucus contained 1.5 ± 0.6%DW of carbon and 0.4 ± 0.1%DW of nitrogen, producing a C:N ratio of 3.9 ± 0.4.

**Fig. 1:**
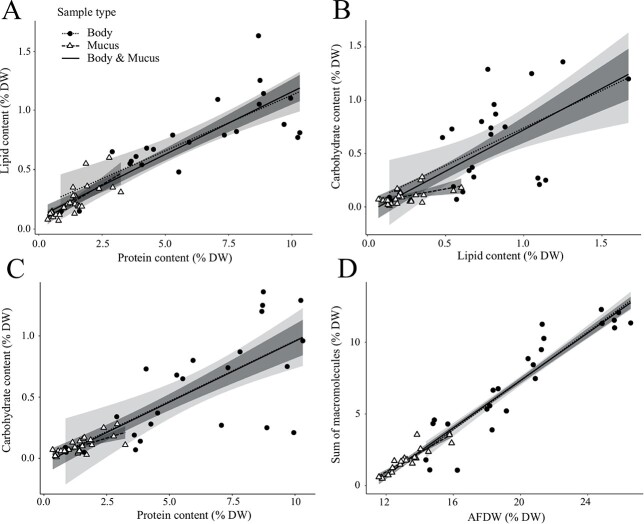
Comparison of the macromolecular content (protein, lipid, carbohydrate; A, B and C) and the sum of macromolecules to the ash-free dry weigh (AFDW, D) of mucus and body tissues in jellyfish species (*A. aurita*, *C. fulgida*, *C. pacifica*, *E. inexplicata* and *R. pulmo*) expressed as percentage of dry wet (%DW) of the samples. The lines represent the linear regressions and the shaded area is the confidence interval (see details in SI, Supplementary Table S2). The solid lines are the linear regressions on the whole data set with (A) lipid (LD) vs protein (PT; LD = 0.10 ± 0.01 PT + 0.10 ± 0.03), (B) carbohydrate (CH) vs lipid (CH = 0.78 ± 0.10 LP—0.05 ± 0.06), (C) carbohydrate vs protein, (CH = 0.10 ± 0.01 PT—0.04 ± 0.05) and (D) sum of macromolecules (SM) vs AFDW (SM = 1.12 ± 0.03 AFDW +11.24 ± 0.16).

Protein, lipid and carbohydrate contents were all linearly correlated with each other, with the type of sample (body or mucus) having no effect on the linear regressions as indicated by the absence of significant interaction (*P* > 0.05; SI, Supplementary Table S2) between the type of tissue and the macromolecule contents (Fig. 1A–C; SI, Supplementary Table S2). The sum of macromolecules was linearly correlated with the AFDW (Fig. 1D; SI, Supplementary Table S2), though there was a consistent offset—with the sum of macromolecules being less than AFDW—as indicated by the negative intercept (slope: 0.8 ± 0.0, intercept = −9.1 ± 0.3; SI, Supplementary Table S2). Both tissue types had the same linear regression between the sum of macromolecules and the AFDW (*P* = 0.20, Fig. 1D; SI, Supplementary Table S2).

**Fig. 2:**
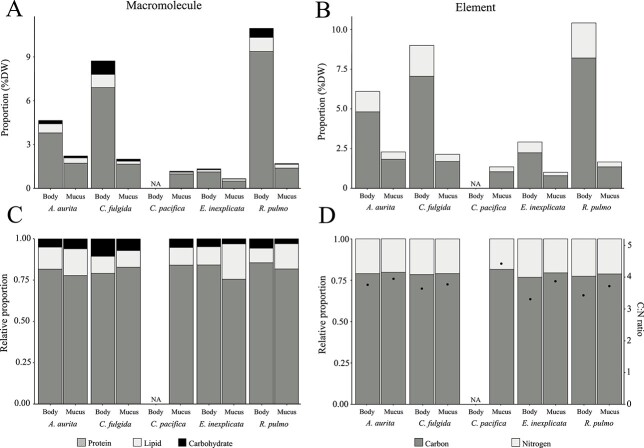
Proportion of proteins, lipids and carbohydrates (A) and carbon and nitrogen (B) in the body and mucus of jellyfish species (*A. aurita*, *C. fulgida*, *C. pacifica*, *E. inexplicata* and *R. pulmo*) expressed as percentage of the dry weight (DW) of the sample and as relative proportion of the total proteins, lipids and carbohydrates (C) and carbon and nitrogen (D) content. The dots represent the C:N ratio (D).

The absolute content of macromolecules (Fig. 2A) and carbon and nitrogen (Fig. 2B) in body tissue and mucus varied widely between species but were consistent when expressed in relative proportion (Fig. 2C, D). Overall, the relative proportions of protein:lipid:carbohydrate and carbon:nitrogen were fairly consistent between the mucus and the body and across the species with an average ratio of 14.6:2.3:1 and 3.7:1, respectively.

**Fig. 3:**
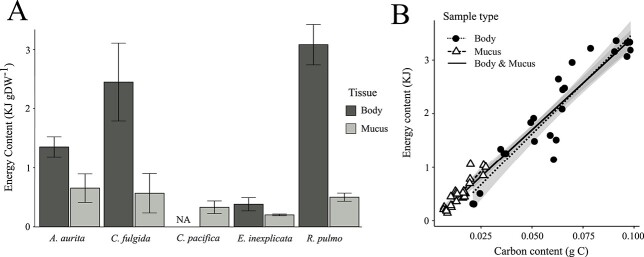
Energy content of the body (dark gray) and mucus (light gray) of jellyfish species (*A. aurita*, *C. fulgida*, *C. pacifica*, *E. inexplicata* and *R. pulmo*) normalized to the dry weight (DW) of the samples (A). Error bars show the standard deviation. Linear regression between the energy content (EC) and the carbon content (CC) of jellyfish body (circles) and mucus (triangles; B). The lines represent the linear regressions and the shaded area is the confidence interval (see details in SI, Supplementary Table S2). The solid line is the linear regressions on the whole data set (EC = 35.03 ± 1.27 CC—0.05 ± 0.06, SI, Supplementary Table S2).

The energy content of the jellyfish body and mucus ranged from 0.4 to 3.1 KJ g^−1^ DW for the body tissue (*A. aurita*: 1.3 ± 0.2, *C. fulgida*: 2.4 ± 0.7, *E. inexplicata*: 0.4 ± 0.1 and *R. pulmo*: 3.1 ± 0.3 KJ g^−1^ DW) and from 0.2 to 0.6 KJ g^−1^ DW for the mucus (*A. aurita*: 0.6 ± 0.2, *C. fulgida*: 0.6 ± 0.3, *C. pacifica*: 0.3 ± 0.1, *E. inexplicata*: 0.2 ± 0.01 and *R. pulmo*: 0.5 ± 0.1 KJ g^−1^ DW). The mucus was consistently less dense in energy than the body tissues (Fig. 3A), with its energy content varying largely with the species (48, 23, 87 and 16% of the energy content of body tissue for *A. aurita*, *C. fulgida*, *E. inexplicata* and *R. pulmo,* respectively). The energy content of jellyfish biomass (body and mucus) was linearly correlated with its carbon content (Fig. 3B), with the type of tissue having no effect on the linear regression (SI, Supplementary Table S2).

## DISCUSSION

Our measurements of the protein and carbohydrate content of the jellyfish body (protein: 6.0 ± 3.0%DW, carbohydrate: 0.6 ± 0.4%DW) are in the range of previous studies (protein: 2.1–28.6%DW, carbohydrate: 0.1–2.9%DW; SI, Supplementary Table S1), whereas our values for the lipid content of the body (0.7 ± 0.4%DW) are slightly lower than previous studies (1.2–11.0%DW; SI, Supplementary Table S1). The high percentage of inorganic material in jellyfish biomass (body: 80.0 ± 3.9%DW, mucus: 87.1 ± 1.5%DW, body and mucus: 83.8 ± 4.6%DW) is likely due to the fact that jellyfish are osmoconformers, having an internal osmolarity similar to their surrounding environment (Kogovšek et al., 2014). When we consider the water content of jellyfish (~96% of wet weight; Pitt et al., 2013), 1 kg of jellyfish would contain 40 g of dried matter and 960 g of pure water. In seawater of salinity 35 g/kg, 960 g of pure water would be associated with 33.6 g of salts. Hence, the potential salt content of jellyfish tissue can explain ~ 84% of the DW of jellyfish body (33.6 g salt in 40 g DW), which matches our measured values. We suggest that the high inorganic content of the mucus can also be explained by its high salt content, as already suggested for gastropod mucus (Stabili et al., 2015).

We observed a notable discrepancy between the sum of macromolecules and the AFDW for both mucus and body tissue (Fig. 1D) which are both representing the organic content of the samples. A possible explanation is that the water bounded to jellyfish DW induces an overestimation of the AFDW (Kogovšek et al., 2014). In addition, nucleic acids were not considered in the sum of macromolecules, thus inducing an underestimation of the macromolecular content. Altogether, the bound water found in jellyfish DW and the absence of nucleic acids in our macromolecular calculations likely caused the sum of macromolecules to be significantly smaller than the AFDW.

Our data suggest that the relative content of macromolecules, and of carbon and nitrogen, is conserved between the jellyfish body and mucus across species. The high proportion of protein in jellyfish bodies (82 ± 4% of macromolecules) reflects that most of the jellyfish body tissue is made of proteinous mesoglea (Lucas, 1994; Kogovšek et al., 2014). In contrast, the high protein content of the mucus (80 ± 4% of macromolecules) was unexpected as the glycoproteins found in mucus, which make up most of the dry content of mucus (~60%; Bakshani et al., 2018), usually have 50–80% of their molecular weight comprised of carbohydrates (Bansil and Turner, 2018). We would hence expect the protein/carbohydrate ratio of the glycoproteins to determine the protein/carbohydrate ratio of the mucus. Our results suggest that the glycoproteins produced by jellyfish are low in carbohydrates. The scarcity of highly soluble hydrocarbon chains in the mucus, as suggested by our data, would decrease its solubility and rigidity (Davies and Viney, 1998) allowing it to hold more to the epithelium, while retaining viscosity.

The high protein content of the mucus is reflected in the relatively low C:N ratio (3.9 ± 0.4), which contrasts with the higher but largely variable C:N ratio previously found for the mucus of the scyphomedusa *Chrysaora quinquecirrha* (C:N = 8.1 ± 6.2, Condon et al., 2011). The difference in mucus elemental C:N ratio between Condon et al. (2011) and our study is most likely caused by a difference in the analyzed material. We analyzed concentrated mucus directly extracted from stressed jellyfish (including the particulate and dissolved phases), whereas Condon et al. (2011) studied the dissolved organic phase of the mucus produced by unstressed jellyfish. The two differing results indicate that the dissolved organic phase of the mucus is less rich in nitrogen compounds (e.g. lacking proteins and amino acids) compared with the particulate organic phase. In addition, the ammonium present in the concentrated mucus would lower the C:N ratio of the mucus. Although it has been suggested that jellyfish mucus produced under different conditions or situations (e.g. reproduction, feeding, stress) might cause differences in biochemical composition (Tinta et al., 2021), there is to date no evidence to confirm this hypothesis.

## ECOLOGICAL IMPLICATIONS

The capacity of marine ecosystems to support stocks of living resources can be estimated by calculating energy flux circulating through food webs (Schaafsma et al., 2018). Our calculations of energy density based on macromolecular composition of jellyfish body biomass (0.4–3.1 KJ g^−1^ DW, Fig. 3) are slightly lower than in a previous study (2.83–4.30 KJ g^−1^ DW, Doyle et al., 2007). The energy content of jellyfish bodies is low compared with most marine organisms (e.g. fishes: 14.8–39.3 kJ g^−1^ DW, crustaceans: 7.1–25.3 kJ g^−1^ DW, squid: 16.2–24.0 kJ g^−1^ DW; Schaafsma et al., 2018) owing to their high inorganic content. Despite their low energy content, recent studies have shown that jellyfish are consumed by many organisms throughout the marine food chain (Marques et al., 2019). Their high water content allows jellyfish biomass to be quickly digested (e.g. up to 20 times faster than shrimps) thus counterbalancing their low energy density and reaching comparable rates of energy acquisition for predators feeding on fish or crustaceans (Hays et al., 2018). In addition, the consumption of gelatinous biomass by fishes can represent an alternative food resource when primary prey are not available (Briz et al., 2018) allowing fish to adapt to prey availability. Furthermore, the dietary value of jellyfish is enhanced by their high abundance, their slow movements (no need of active pursue) and fast growth rates. Feeding on jellyfish could thus be strategically beneficial, especially when energy-rich tissue, such as gonads and arms, are consumed preferentially (Hays et al., 2018).

The lower energy density of jellyfish mucus (0.2 to 0.6 KJ g^−1^ DW, Fig. 3A) compared with the body (0.4–3.1 KJ g^−1^ DW, Fig. 3A) can be explained by its higher water and thus salt content. When compared with carbon content (Fig. 3B), the energy content of the mucus and the body remain proportionate due to the similar proportion of macromolecules, providing a convenient relationship to calculate the energy content of jellyfish biomass based on carbon content.

Our jellyfish organic matter (body and mucus) was rich in nitrogen (C:N = 3.7) compared with the global medians (6.6–7.4, Martiny et al., 2014) and to other marine zooplankton organisms (4.8–6.2 for crustacean zooplankton; Pitt et al., 2013). Jellyfish, particularly in high abundances such as during blooms, represent a storage of nitrogen-rich organic matter that can be supplied to the environment through excretion of inorganic nutrients (Hubot et al., 2021) and mucus production (Condon et al., 2011) or reach higher trophic levels through predation (Hays et al., 2018). Subsequently, when a jellyfish dies and starts decaying, its body mass will be available for bacterial degradation. The microbial remineralization of jellyfish organic matter (mucus and carcasses) may have an important impact on nutrient cycles (Condon et al., 2011; Tinta et al., 2021) supplying primary producers with nutrients and ultimately supporting the whole food chain. As nitrogen availability limits primary productivity in most of the surface ocean (~75%; Bristow et al., 2017), the remineralization of the labile nitrogen-rich jellyfish organic matter could potentially reduce nitrogen limitation, thus enhancing primary production in nitrogen-limited environments. In addition, the sinking of carcasses creates a downward flux of organic nutrient, participating in carbon sequestration and supplying the deep-sea food webs with organic matter thus supporting commercially important invertebrate species (Dunlop et al., 2017). Overall, jellyfish organic matter has the potential to support the marine food web at multiple levels.

## CONCLUSIONS

Our study provides a first general characterization of the biochemical composition of jellyfish mucus across different species and highlights its similarity with the jellyfish body composition. The data suggest that jellyfish organic matter is not species-specific and indicate a much higher homogeneity in jellyfish organic matter than previously expected (Tinta et al., 2021). As jellyfish biomass can largely exceed (up to three times) the biomass of fish in highly productive ecosystems (Lynam et al., 2006), it is crucial to investigate its impact on marine ecosystem productivity. Our data facilitate the inclusion of jellyfish in ecosystem studies by providing convenient and valuable biochemical relationships allowing to model the role of jellyfish in marine food webs and biogeochemical cycles and thus to estimate changes in the future ocean.

## Supplementary Material

Supplementary_Information_Hubot_et_al_2022_fbab091

Hubot_et_al_2022_data_fbab091
