# Supplementary material for: Similarities between the biochemical composition of jellyfish body and mucus
Source: J Plankton Res. 2022 Jan 21;44(2):337–44. doi: 10.1093/plankt/fbab091 (PMC8962712; doi:10.1093/plankt/fbab091)
Supplement: Supplementary_Information_Hubot_et_al_2022_fbab091 [file supplementary_information_hubot_et_al_2022_fbab091.docx]

Supplementary Information

# Literature review

**Table S1**. Review of the biochemical composition (protein, lipid and carbohydrate) of members of marine zooplankton as a percentage of dry body weight, and jellyfish mucus as a relative percentage.

| Species | Location | Protein (%DW) | lipid (%DW) | carbohydrate (%DW) | Reference |
| --- | --- | --- | --- | --- | --- |
| **Gelatinous zooplankton** |  |  |  |  |  |
| All | Southern Ocean | 7.5–17.0 | 1.8−4.6 | 0.4−1.7 | Clarke et al., 1992 |
| **Jellyfish** |  |  |  |  |  |
| *Aurelia aurita* | Baltic sea | 5.9 | 2.17 | 2.9 | Scheider, 1988 |
| *Aurelia aurita* | Southampton | 2.07 - 28.56 | 1.17 - 11 | 0.10-1.12 | Lucas, 1994 |
| *Atolla wyvelli* | Antartic | 16.95 | 4.17 | 1.71 | Clarke et al., 1992 |
| *Rhizostoma pulmo* | Mediterranean | 8.6 | 3.5 | - | Ceccaldi et al.,1978 |
|  |  |  |  |  |  |
| **Copepods** |  |  |  |  |  |
| all copepods |  | 24 - 82 | 2 - 73 | 0.2-5.1 | Båmstedt, 1986 |
| *Pseudodiaptomus annandalei* | Taiwan | 57 | 16 |  | Rayner et al. 2015 |
|  |  |  |  |  |  |
| **Zooplankton** |  |  |  |  |  |
| All | Kerguelen Islands | 21.5 | 8.9 | 3.2 | Harmelin-Vivien et al., 2019 |
|  |  |  |  |  |  |
| **Mucus** |  | (relative %) | (relative %) | (relative %) |  |
| *Aurelia aurita* | Gulf of Aqaba | 73 | 27 | 5 | Ducklow & Mitchell, 1979 |

References:

Båmstedt, U. (1986). Chemical composition and energy content. In: Corner, E. D. S., O'Hara, S. C. M. (eds.) *The biological chemistry of marine copepods*. Oxford University Press, New York, 1–58

Ceccaldi, H. J., A. Kanazawa, and S-I. Teshima. 1976. Chemical composition of some Mediterranean macroplanktonic organisms. *Tethys,* **8**, 295–298.

Clarke A, Holmes LJ, Gore DJ (1992) Proximate and elemental composition of gelatinous zooplankton from the Southern Ocean. *J. Exp. Mar. Biol. Ecol*., **155**, 55-68.

Ducklow, H. W. and Mitchell, R. (1979) Composition of mucus released by coral reef coelenterates1. *Limnol. Oceanogr.*, **24**, 706–714.

Harmelin-Vivien M, Bӑnaru D, Dromard CR, Ourgaud M, Carlotti F (2019) Biochemical composition and energy content of size-fractionated zooplankton east of the Kerguelen Islands. *Polar Biol*, **42**, 603–617.

Lucas, C. H. (1994) Biochemical composition of *Aurelia aurita* in relation to age and sexual maturity. *J. Exp. Mar. Biol. Ecol.*, **183**, 179–192.

Rayner TA, Jørgensen NOG, Blanda E, Wu C-H, Huang C-C, Mortensen J, Hwang J-S, Hansen BW (2015) Biochemical composition of the promising live feed tropical calanoid copepod *Pseudodiaptomus annandalei* (Sewell 1919) cultured in Taiwanese outdoor aquaculture ponds. *Aquaculture*, **441**, 25–34.

Schneider G (1988) Chemical composition and biomass parameters of the common jellyfish *Aurelia aurita*. *Helgolander Meeresunters* **42**, 319–327.

# Material and methods

## Lipid analyses

Total lipid of the samples were extracted using a single step extraction method relying on the chloroform-methanol solvent system, following the protocol by Axelsson and Gentili (2014; see protocol below). Briefly, 10 mg of freeze-dried tissue was homogenized with a 2∶1 chloroform-methanol (v/v) mixture to a final volume of 3 mL and vortexed for 30 seconds. 1 mL of Ultra-pure water was then added to the solution, which was then vortexed briefly and centrifuged to 1000 g for 10 minutes. Subsequently, supernatant was removed along with the cell debris and 1 mL of the lower phase was collected and transferred to vial for nitrogen drying at 60°C. The lipid amount was measured using a charring method (Marsh and Weinstein 1966; see protocol below) by adding 500 µL of H_2_SO_4_ and incubating 15 min at 200°C. After cooling down, 3 mL of water was added and lipid concentration was determined using a UV-Vis spectrophotometer at 375 nm. The assay was calibrated with known amounts of cholesterol (Sigma Aldrich, C8503; see table of dilutions in protocol).

### Protocol:

**Extraction**: Single step method (based on Axelsson and Gentili 2014)

1. Weight 10 mg freeze-dried tissue, add to 8 mL glass vials
2. Make standards into 8mL glass vials for a final volume of 1 mL
3. Add 2 and 3 mL of 2:1 chloroform:methanol to standards and samples, respectively.
4. Vortex for 30 sec.
5. Add 1 ml of 0.73 % w/v NaCl solution (for standards) or 1 mL of pure water (for samples)
6. Vortex briefly
7. Centrifuge to 1000 g for 10 min
8. Remove supernatant and cells debris
9. Collected 1 ml of the lower phase
10. Transfer to a small vial and dry at 60°C with nitrogen

**Measurement**: Charring method (based on Marsh and Weinstein 1966)

1. Start heating the aluminium plate (320°C) and check to temperature with the laser until reaching 200°C
2. Add 500 µl of H_2_SO_4_ to the vials and mix gently (use face shield)
3. Place vials in the aluminium heating block for 15 min at 200°C
4. Cool the vial in water at room temperature and transfer to cold aluminium block (from fridge)
5. Add 3 ml of water, mix and let it cool down
6. Mix well before transferring 1 ml to semi-micro cuvettes and read at **375 nm**

Table of cholesterol dilutions for calibration:

Stock solution: 20 mg of Cholesterol to 100 ml of 2:1 chloroform:methanol = 200 mg/L

| Standard | Volume of Stock  (µl) | Volume 2:1 Chloro:Methanol  (µl) | Final concentration (mg/l) |
| --- | --- | --- | --- |
| 1 | 0 | 1000 | 0 |
| 2 | 200 | 800 | 40 |
| 3 | 400 | 600 | 80 |
| 4 | 600 | 400 | 120 |
| 5 | 800 | 200 | 160 |
| 6 | 1000 | 0 | 200 |

## Protein analyses

Total protein concentration in samples was measured using a modification of the assay by Lowry (Lowry et al. 1951; Gerhardt et al. 1994). Briefly, approximatively 20 mg freeze-dried tissue were dissolved into 2 mL of ultra-pure water (≈10 mg/mL). 100 µL of the sample solution was diluted to a final volume of 500 µL, mixed with the Lowry solution and incubated for 20 minutes in the dark at room temperature. Samples were then incubated again in the same conditions for minimum 30 minutes with 0.1 mL of Folin & Ciocalteu’s phenol reagent 1N (Sigma Aldrich, F9252). Protein concentration was determined using a UV-Vis spectrophotometer at 750 nm, calibrated against bovine serum albumin (Sigma Aldrich, A3059; see table of dilution in protocol).

### Protocol:

1. Weight approximatively 20 mg freeze-dried tissue, add to 8 mL glass vials with 2 ml of water (≈10 mg/mL)
2. Vortex for 30 sec.
3. Prepare a stock solution of BSA and the Lowry solution by mixing 500 µL of 1% CuSO_4_ and 500 µL of 2% KNaC_4_H_4_O_6_4H_2_O, then add 50 mL of 2% NaCO_3_ in NaOH
4. Transfer 100 µL of sample to a 8mL glass vial + 400 µL of water

500 µL of standard to an 8mL glass vial

1. Add 0.7 mL of Lowry Solution
2. Cap and vortex briefly at low speed
3. Incubate for 20 min in the dark at room temperature
4. After 15 min of incubation, prepare the Folin reagent dilution (1:1 Folin:water)
5. After 20 min of incubation, add 0.1 mL of diluted Folin reagent
6. Cap and vortex immediately
7. Incubate for 30 min or longer in the dark at room temperature
8. After 30 min, vortex briefly and transfer 1 mL to a semi-micro cuvette and read at **750 nm**

Table of BSA dilutions for calibration:

Stock solution: 50 mg of BSA to 250 ml of water = 200 mg/L

| Standard | Volume of Stock  (mL) | Volume of water  (mL) | Final concentration (mg/l) |
| --- | --- | --- | --- |
| 1 | 0 | 10 | 0 |
| 2 | 2 | 8 | 40 |
| 3 | 4 | 6 | 80 |
| 4 | 6 | 4 | 120 |
| 5 | 8 | 2 | 160 |
| 6 | 10 | 0 | 200 |

## Carbohydrate analyses

Total carbohydrate concentrations were measured using the Dubois assay (DuBois *et al.*, 1956). 500 µL of 5% w/v phenol (ref) was mixed with 1 mL of the sample solution used for the protein assay (≈10 mg/mL). 2.5 mL of H_2_SO_4_ was then added to the mixture and mix gently. After a minimum of 20 min standing, samples were vortexed and read at 490 nm against glucose.

### Protocol:

1. Weight approximatively 20 mg freeze-dried tissue, add to 8 mL glass vials with 2 ml of water (≈10 mg/mL)
2. Vortex for 30 sec.
3. Prepare a stock solution of glucose
4. Use 1 ml of sample + 500 µl 5% w/v phenol, mix
5. Add 2.5 ml H_2_SO_4_, mix gently
6. Stand > 20 min
7. Vortex briefly and transfer 1 mL to a semi-micro cuvette and read at **490 nm**

Table of glucose dilutions for calibration:

Stock solution: 25 mg of glucose to 250 ml of water = 100 mg/L

| Standard | Volume of Stock  (mL) | Volume of water  (mL) | Final concentration (mg/l) |
| --- | --- | --- | --- |
| 1 | 0 | 10 | 0 |
| 2 | 2 | 8 | 20 |
| 3 | 4 | 6 | 40 |
| 4 | 6 | 4 | 60 |
| 5 | 8 | 2 | 80 |
| 6 | 10 | 0 | 100 |

# Results

## Linear regressions

**Table S2.** Summary of linear regression (~) and ANCOVA (:). SM = sum of macromolecules, AFDW = ash-free dry-wet, EC = energy content, CC = carbon content.

| Relation | Tissue | slope | SD | Intercept | SD | n | R² | p |
| --- | --- | --- | --- | --- | --- | --- | --- | --- |
| Lipid ~ Protein | body | 0.09 | 0.01 | 0.19 | 0.10 | 24 | 0.61 | *** |
|  | mucus | 0.13 | 0.02 | 0.05 | 0.04 | 27 | 0.53 | *** |
|  | both | 0.10 | 0.01 | 0.10 | 0.03 | 51 | 0.80 | *** |
| Protein:Tissue | both | - | - | - | - | - | - | 0.45 |
| Carbohydrate ~ Protein | body | 0.10 | 0.02 | -0.03 | 0.15 | 24 | 0.46 | *** |
|  | mucus | 0.06 | 0.01 | 0.01 | 0.02 | 27 | 0.48 | *** |
|  | both | 0.10 | 0.01 | -0.04 | 0.05 | 51 | 0.67 | *** |
| Protein:Tissue | both | - | - | - | - | - | - | 0.54 |
| Carbohydrate ~ Lipid | body | 0.70 | 0.21 | 0.04 | 0.17 | 24 | 0.31 | ** |
|  | mucus | 0.30 | 0.09 | 0.03 | 0.02 | 27 | 0.24 | ** |
|  | both | 0.78 | 0.10 | -0.05 | 0.06 | 51 | 0.56 | *** |
| Lipid:Tissue | both | - | - | - | - | - | - | 0.28 |
| SM ~ AFDW | body | 1.00 | 0.05 | 12.60 | 0.40 | 72 | 0.85 | *** |
|  | mucus | 1.21 | 0.07 | 11.09 | 0.15 | 66 | 0.81 | *** |
|  | both | 1.12 | 0.03 | 11.24 | 0.16 | 139 | 0.93 | *** |
| AFDW:Tissue | both | - | - | - | - | - | - | 0.20 |
| EC ~ CC | body | 38.05 | 3.05 | -0.28 | 0.20 | 24 | 0.87 | *** |
|  | mucus | 40.58 | 3.61 | -0.09 | 0.06 | 27 | 0.83 | *** |
|  | both | 35.03 | 1.27 | -0.05 | 0.06 | 51 | 0.94 | *** |
| AFDW:Tissue | both | - | - | - | - | - | - | 0.78 |

# References

Axelsson, M. and Gentili, F. (2014) A Single-Step Method for Rapid Extraction of Total Lipids from Green Microalgae. *PLOS ONE*, **9**, e89643.

DuBois, Michel., Gilles, K. A., Hamilton, J. K., Rebers, P. A., and Smith, Fred. (1956) Colorimetric Method for Determination of Sugars and Related Substances. *Anal. Chem.*, **28**, 350–356.

Gerhardt, P., Murray, R. G. E., Krieg, N. R., and Wood, W. A. (1994) *Methods for General and Molecular Bacteriology*. American Society for Microbiology.

Lowry, O. H., Rosebrough, N. J., Farr, A. L., and Randall, R. J. (1951) Protein measurement with the Folin phenol reagent. *J Biol Chem*, **193**, 265–275.

Marsh, J. B. and Weinstein, D. B. (1966) Simple charring method for determination of lipids. *J. Lipid Res.*, **7**, 574–576.
